# Prestalk-like positioning of de-differentiated cells in the social amoeba *Dictyostelium discoideum*

**DOI:** 10.1038/s41598-024-58277-3

**Published:** 2024-04-01

**Authors:** Yuka Shirokawa, Masakazu Shimada, Nao Shimada, Satoshi Sawai

**Affiliations:** 1 Graduate School of Arts and Sciences, University of Tokyo, Tokyo, 153-8902 Japan; 2 Research Center for Complex Systems Biology, Universal Biology Institute, University of Tokyo, Tokyo, 153-8902 Japan

**Keywords:** Cooperation, Division of labor, Developmental stability, Multicellularity, Evolutionary theory, Social evolution

## Abstract

The social amoeba *Dictyostelium discoideum* switches between solitary growth and social fruitification depending on nutrient availability. Under starvation, cells aggregate and form fruiting bodies consisting of spores and altruistic stalk cells. Once cells socially committed, they complete fruitification, even if a new source of nutrients becomes available. This social commitment is puzzling because it hinders individual cells from resuming solitary growth quickly. One idea posits that traits that facilitate premature de-commitment are hindered from being selected. We studied outcomes of the premature de-commitment through forced refeeding. Our results show that when refed cells interacted with non-refed cells, some of them became solitary, whereas a fraction was redirected to the altruistic stalk, regardless of their original fate. The refed cells exhibited reduced cohesiveness and were sorted out during morphogenesis. Our findings provide an insight into a division of labor of the social amoeba, in which less cohesive individuals become altruists.

## Introduction

The social amoeba *Dictyostelium discoideum* is a soil-living eukaryote with facultative sociality under non-nutrient conditions^1^. Cells grow and divide in the presence of nutrient sources, such as bacteria. However, when starved, they aggregate to form a multicellular slug that culminates in a fruiting body composed of differentiated spores and stalk cells. *Dictyostelium* has served as a model microorganism to study cellular cooperation^2^, since death of stalk cells that support the spores^1^ can be viewed as altruistic behavior. Cheating that exploits benefits of the stalk investment at the cost of others has been extensively researched, and nature has found ways to reduce the deleterious effects of the cheating through keeping cooperator cells at close distances^3,4^, kin recognition using cell-surface proteins^5^, and suppression of the sporulation of the cheaters^6^. Besides the cheating, the multicellularity is also likely to be vulnerable to environmental changes that raise fitness of a cell as a solitary individual rather than as a multicellular group member. However, it is interesting to note that the multicellularity of the social amoeba exhibits developmental stability in the face of transient environmental changes; after 4 to 6 h of starvation onward, cells become engaged in the differentiation process^7^ and no longer quickly revert to the growth phase, even with a renewed nutrient supply^8^. Instead, cells continue forming fruiting bodies and complete the process within 24 h. The inability of differentiating cells to switch back to solitary growth (hereafter referred to as social commitment) depends on the multicellular context. When cells that are mechanically dissociated are replenished with nutrients, they revert to solitary growth^7–9^. Thus, unless mechanical dissociation, the only way for cells to return to a solitary state is to mature into spores and then germinate, which is a long process that takes a few days. The benefit of the social commitment is puzzling, especially given that the social amoeba colonizes sites with a frequent supply of rich dung or leaf mold^10^, which favor quick reversion to solitary growth^11^. Existence of a phagocytic cell type that removes intruding bacteria within a slug^12^ suggests that it is not unusual for socially committed cells to be exposed to nutrients. To understand the multicellular basis that maintains the social commitment in *Dictyostelium*, we studied the outcomes of premature de-commitment. In our experiments, cells were forced to take the dedifferentiation path by short-term refeeding, and their fate when mixed with socially committed cells was traced using live-cell imaging.

## Results

### Forced refeeding experiments

**Figure 1:**
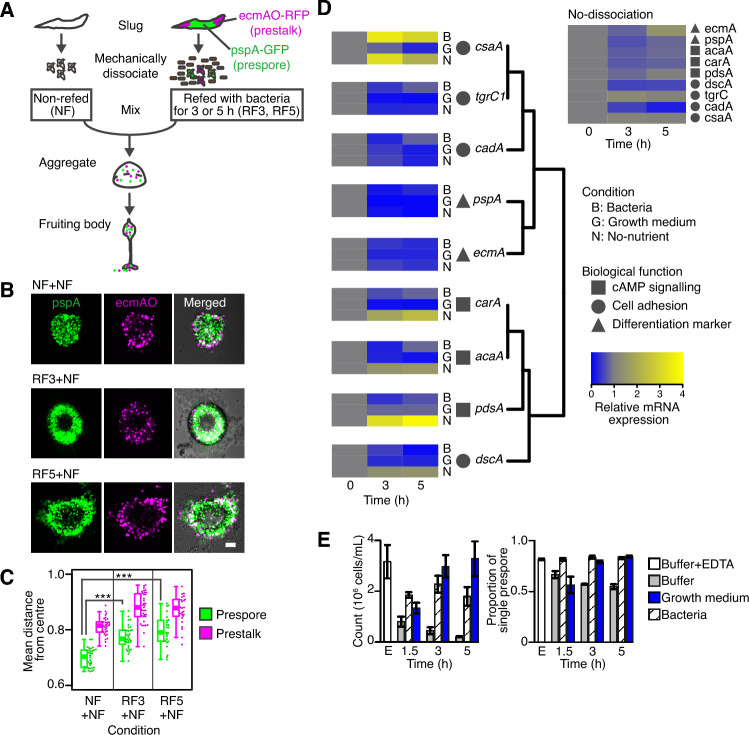
Prestalk-like positioning and cell-state of refed cells. (**A**) A schematic of refeeding and reaggregation of a cell mixture. Dissociated cells were shaken with bacteria for 3 or 5 h (Refed: RF3, RF5), washed and mixed with non-refed cells (Non-refed: NF). Refed (RF) cells harbored prespore (pspA-GFP, green) and prestalk (ecmAO-RFP, magenta) markers. (**B**) Representative images of mixed aggregates 1.5–2 h after plating (pspA: GFP-channel, ecmAO: RFP-channel, Merged: Bright-field and fluorescence images). ‘NF + NF’: Mixture of NF cells with and without the cell-type markers as a control condition. ‘RF3 + NF’ and ‘RF5 + NF’: Mixture of NF cells (without the cell-type markers) and RF3 or RF5 cells (with the cell-type markers), respectively at RF:NF = 15:85 ratio. A scale bar = 50 µm. (**C**) Normalized mean distance between the aggregate center and cells of prestalk or prespore origin. The sample size N (biological replicate, aggregates): NF + NF = (4, 34), RF3 + NF = (4, 35), RF5 + NF = (3, 25). Circles: Values for individual aggregates. Lines inside of box plots: The median values. ***P < 0.0001 by *t* test. (**D**) qRT-PCR analysis. ‘B’: Gene expression levels of dissociated cells in bacterial suspension, ‘G’: growth medium, ‘N’: non-nutrient buffer (left panel). ‘No-dissociation’: Unperturbed slugs 18 h into starvation onward (upper right panel). Gene expression levels were normalized by the 0 h point. The sample size N = 3 for biological replicates. (**E**) Cell cohesiveness of RF and NF cells. The number of single cells within a sample (left panel). The proportion of single prespore cells in the total single cells within a sample (right panel). ‘E’: Cells in the phosphate buffer containing EDTA (Buffer + EDTA) as a non-adhesive control. ‘Buffer’ corresponds to the NF condition, and 'Growth medium’ and ‘Bacteria’ represent the RF condition. The number (1.5, 3, and 5) indicates hours shaken in suspensions. The sample size N = 3 for biological replicates, with 380–5966 cells per condition. Error bars: Standard error.

We utilized a unique property of the social amoeba development, where mechanically dissociated cells replated on agar without nutrients rapidly reaggregate and recapitulate their respective developmental stages within a few hours^13^. For the de-commitment, dissociated cells from the slug stage were fed with bacteria for 3 or 5 h (hereafter referred to as RF3 and RF5 cells, respectively). The refed cells (RF cells) were then allowed to reaggregate with cells that were not refed (NF cells, socially committed) (Fig. 1A). The cell mixture jointly formed a tight mound within 1.5–2 h after plating (Supplementary Fig. S1, Movie S1, S2). In the mixture of NF cells with and without the cell-type marker for prestalk and prespore (NF + NF), prestalk cells were found in the peripheral region of the aggregate, whereas prespore cells were more uniformly distributed (Fig. 1B, NF + NF) in line with the previously described sorting pattern^14^. In contrast, in the mixture of RF and NF cells, RF cells (with the cell-type marker, fluorescent cells) were positioned at the periphery regardless of the original cell type (Fig. 1B, RF3 + NF, RF5 + NF), whereas NF cells (without the cell-type marker, non-fluorescent cells) were more abundant in the aggregation center. The statistical analysis confirmed the significant impact of the refed treatment on cell positioning (Fig. 1C: Overall model: GLM and analysis of deviance, cell fate: *P* < 0.0001, treatments: *P* < 0.0001). For the detailed statistical analysis, see Supplementary Table S1. The swap control exhibited a consistent pattern (Supplementary Fig. S2A, RF(Fl−) + NF(Fl+)), indicating that the marker gene expression itself did not affect the sorting pattern. Furthermore, our supporting data (Supplementary Fig. S2B,C) indicates that the segregation was a relative behavior when RF cells interacted with the NF cells, dependent on nutrient replenishment.

To characterize the cell state after nutrient replenishment, we conducted quantitative real-time polymerase chain reaction analysis (real-time PCR), targeting genes associated with differentiation, adhesion, and aggregation (Table S2). The results showed reduced expression of differentiation marker in RF cells (Fig. 1D, *pspA*, *ecmA*), indicating partial dedifferentiation (Fig. 1D; Bootstrap test, adjusted* P* > 0.025). Although genes encoding the main adhesion molecules (*cadA*, *csaA*, and *tgrC1*)^15^ showed no nutrient-specific changes, *carA*, *acaA*, *pdsA,* and *dscA* genes (Fig. 1D) exhibited reduced expression by refeeding and induction in a non-nutrient buffer (Fig. 1D; GLM and analysis of deviance, adjusted* P* < 0.05). *CarA*, *acaA*, and *pdsA* are essential for the cAMP relay during cell aggregation, and *dscA* (discoidin I) is implicated in cell-substratum adhesion during aggregation^16^. RF cells initially appeared well mixed in the aggregate and were subsequently sorted (Supplementary Fig. S1, Movie S2), thus the observed sorting pattern is unlikely to result from delayed chemotaxis due to the reduced reaggregation gene expression (Fig. 1D). RF cells were sorted to the periphery and then to the base of the mound (Supplementary Fig. S3A), in contrast to a sorting mechanism involving a tip-forming prestalk subtype^17^. These observations point to an alternative mechanism based on differential adhesion^18^, where cells in the peripheral region of a cell mass should be less cohesive than those in the inner region.

To understand the relationship between differential adhesion and the sorting pattern, we investigate the ability of RF cells to associate with aggregates using adhesion assays^19^. A suspension of mechanically dissociated cells was shaken to facilitate cell agglutination for 1.5, 3, and 5 h, and the number of unattached single cells was quantified (Fig. 1E, left). As a non-adhesive control, phosphate buffer containing EDTA (Buffer + EDTA) was used, as most cells remained unattached. In the NF condition, cells were shaken in plain phosphate buffer, while in the RF condition, they were shaken in phosphate buffer with bacteria or the growth medium. Cells in the bacterial suspension and in growth medium exhibited lower cohesion than cells in the phosphate buffer (Fig. 1E left; GLM and analysis of deviance, adjusted* P* < 0.0001). This indicates lower cohesion of RF cells than NF cells as expected^20^. In addition, the ratio of prespore cells among unattached cells in the phosphate buffer (55%) was lower than that in the non-adhesive control (82%) (Fig. 1E, right). This indicates greater cohesion of NF prespore cells than NF prestalk cells, consistent with the literature^21^. On the other hand, RF prespore cells showed lower cohesion, similar to prespore cells in the non-adhesive control (Fig. 1E right; GLM and analysis of deviance, adjusted *P* = 0.572). Together, the reduced cohesiveness in RF cells compared to NF cells (Fig. 1E, left), along with lower cohesiveness of NF prestalk cells than NF prespore cells (Fig. 1E, right), is consistent with peripheral positioning of less cohesive cells in aggregation (Fig. 1B,C), in line with the differential adhesion hypothesis^18^.

**Figure 2:**
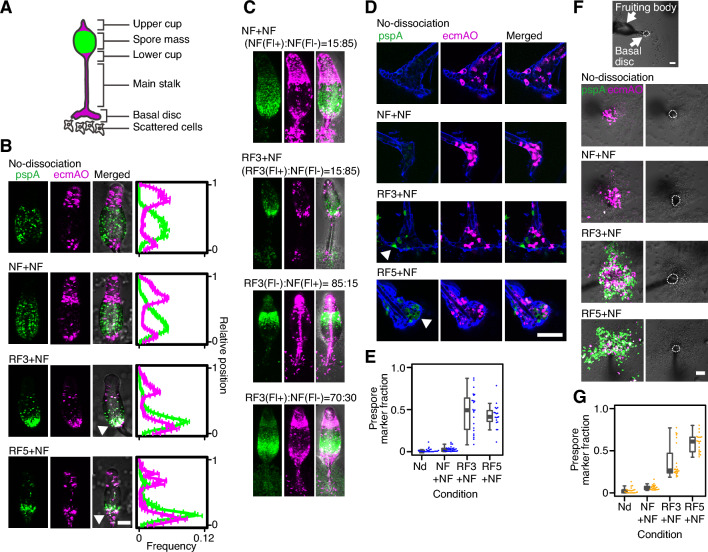
Refeeding redirects cells to the role of prestalk subtype. (**A**) Schematic illustration of the fruiting body. (**B**) Snapshots of the upper region of early fruiting bodies (left panel). Frequency of prestalk (magenta line) and prespore (green) marker-positive pixels along the anterior–posterior axis (right panel). No-dissociation (Nd): A fruiting body formed by unperturbed cells. In RF3 + NF and RF5 + NF, RF cells were found exclusively in the lower cup (arrowheads). The sample size N (biological replicate, fruiting body number): N: Nd = (2, 14), NF + NF = (3, 24), RF3 + NF = (3, 20), RF5 + NF = (3, 13). For abbreviations, see also Fig. 1. (**C**) Early fruiting bodies consisted of different ratios of RF3 and NF cells. Fl+, Fl−: With/without cell-type markers, respectively. (**D**) Fluorescent images of the basal disc. Images of stalk cellulose staining (blue, calcofluor white) were merged with fluorescence images (pspA, ecmAO). Note RF cells of prespore origin are found in the basal disc (RF3 + NF and RF5 + NF) (arrowheads). (**E**) The ratio of prespore marker-positive to prestalk region in the basal disc. N: Nd = (3, 26), NF + NF = (3, 30), RF3 + NF = (3, 23), RF5 + NF = (3, 19). (**F**) Representative images of the basal region of fruiting bodies. Note RF cells (fluorescent cells, left panel) scattered around the basal discs (white circles, right panel) in RF3 + NF and RF5 + NF. (**G**) The ratio of prespore marker-positive region to prestalk region in the solitary cells scattered around the basal discs. N: Nd = (3, 23), NF + NF = (3, 23), RF3 + NF = (3, 23), RF5 + NF = (3, 21). All scale bars = 50 µm. Error bars: Standard error.

Positional bias was evident in the stalk region during culmination. In addition to the main stalk, the stalk cells comprise three types: Upper and lower cups, and basal disc^1^ (Fig. 2A). The normal finger-like form with a prespore cell mass between the upper and lower cups was observed in NF cells (Fig. 2B, NF + NF; Supplementary Fig. S3B, Movie S3). In contrast, in RF + NF, both prespore and prestalk RF cells were abundant in the lower cup region (Fig. 2B, RF3 + NF, RF5 + NF; Supplementary Fig. S3B, Movie S4). Later, RF cells formed the lower cup and basal disc, but tended to be less biased in the upper cup and main stalk (Fig. 2C, RF3 + NF). When RF cells were the majority, RF cells were found in the lower portion of the spore mass and the upper cup in addition to the lower cup and the stalk (Fig. 2C, RF3(Fl+):NF(Fl−) = 70:30).

In the terminal differentiation (8–10 h after plating), the ectopic positioning of RF cells was most notable in the basal disc region, which consists mainly of secreted cellulose and vacuolating non-viable cells^22^. In the controls (‘No-dissociation’, NF + NF), the basal disc was occupied by cells of prestalk-origin (Fig. 2D,E) as expected^23^. On the other hand, in RF + NF (Fig. 2D,E), both prestalk and prespore RF cells were found in the basal disc (Fig. 2D,E, NF + NF versus RF5 + NF: *t*-test, adjusted *P* < 0.0001). In addition, we observed solitary cells scattered around the base of the fruiting body^24^, which appeared to remain at the base when the spore mass was elevated (Movie S3, S4). The solitary cells were amoeboids and displayed random movement (Movie S5), suggesting escaped death as stalks. In the controls, the solitary cells originated from prestalk cells (Fig. 2F,G, ‘No-dissociation’, NF + NF), whereas in RF + NF (Fig. 2F,G, RF3 + NF, RF5 + NF), RF cells of both prespore and prestalk origins were found (Fig. 2F,G: NF + NF versus RF5 + NF: *t*-test, adjusted *P* < 0.0001). We also explored cell fate allocation during terminal differentiation in NF + NF, RF3 + NF, and RF5 + NF conditions. The results suggest that a higher proportion of RF cells tended to solitary cells compared to NF cells, whereas NF cells differentiated into a higher proportion of spores than RF cells (For a detailed explanation and limitations of interpretation, see Supplementary Fig. S4). These results (Supplementary Fig. S4, Movie S5) indicate that the survivors after the terminal differentiation consisted of solitary cells (amoeba) and social cells (spores). Taken together, refeeding experiments (Figs. 1, 2) revealed that RF cells tended to bias toward the stalk region (lower cups and basal disc) and remain on the outside of a fruiting body when interacting with NF cells. Difference in cell–cell cohesiveness between RF and NF cells (Fig. 1E, left) is a possible key factor to explain the observed positioning.

## Discussion

Our results suggest that dedifferentiating *Dictyostelium* cells take a prestalk-like cell state through interactions with differentiating cells (Figs. 1, 2). The system may penalize the premature de-commitment through a division of labor, in which less cohesive cells become altruists. Unlike policing, which usually incurs inevitable costs^25^, the penalization is likely to occur naturally through fruiting body formation, and the social amoeba does not seem to pay explicit costs. The maintenance of the social commitment in *Dictyostelium* has been a long-standing question since the observation was first reported^8^. Our experimental results suggest a cellular dynamics-based mechanism. The positioning of refed cells at the mound periphery resembling prestalk cells (Fig. 1B) can be explained by the mechanism of cell segregation through differential adhesion^18^ between non-refed and refed cells (Fig. 1E). The observed segregation pattern (Fig. 1B) contrasts with a mutant lacking a gene for the cell adhesion protein csA, which becomes spores within wild-type cells^26^. While the mutant cells become spores on agar^26^, they fail to enter aggregates on soil^27^, highlighting the importance of balanced cell–cell and cell-substratum adhesion for proper positioning. Interestingly, the segregation pattern of the defective mutant in cell-substrate adhesion^28^ closely resembles that of the refed cells (Figs. 1, 2), specifically at the mound periphery and lower cup. Although refed cells showed decreased *dscA* gene expression (Fig. 1D) possibly leading to reduced cell-substratum adhesion^16^, refed cells maintained a similar level of cell-substratum adhesion as they could develop on their own (Supplementary Fig. S2B). Our results were obtained from the laboratory clone AX4; whether the same results would hold in wild clones is a future study.

During terminal differentiation, refed cells formed the lower cup (Fig. 2B) and the basal disc (Fig. 2D). While these are recognized terminal states of prestalk cells, the mechanisms underlying cell positioning remain unclear. The localization of refed cells differs from that of the primary stalk cells but is similar to that of the ‘anterior-like cells’^29^, which represent the basal disc, lower cup, and upper cup. The absence of refed cells in the upper cup distinguishes it from the anterior-like cells. This may be related to reduced cAMP sensitivity due to decreased *carA* gene expression in the refed cells (Fig. 1D): Low cAMP sensitivity facilitates redifferentiation of prespore cells into lower cup cells and inhibits the positioning of prestalk cells in the upper cup^30^. Interestingly, it may exhibit frequency dependency; when RF cells were the majority, some RF cells were located in the upper cup (Fig. 2C), reminiscent of anterior-like cells.

Furthermore, heterogeneity in nutritional states among cells participating in multicellularity is a crucial factor in determining cell fate^31,32^. Our observations (Figs. 1, 2) may align with earlier observations^32^, which indicated that cells experiencing starvation initially tended to form spores (similar to NF) when mixed with cells that were subsequently deprived of nutrients (similar to RF). Our findings provide insight into the multicellular basis of the social commitment in the social amoeba and warrant further study of its evolutionary consequences.

## Methods

Detailed information is provided in Supplementary Text.

### Refeeding experiments

A strain harboring the prestalk (ecmAO-RFP) and prespore (pspA-GFP) marker genes were constructed from AX4 cells to identify the original cell fate, taking into account the time required for fluorescent proteins to degrade (more than 4–5 h)^33^. To obtain slug-stage cells, vegetative cells were washed with phosphate buffer (PB) and applied to a 1% PB agar plate and incubated for 18 h. PB containing 20 mM EDTA was applied to the slugs, and the cells were mechanically dissociated by repeated pipetting. For refeeding, the dissociated cells were co-suspended with the concentrated *Escherichia coli* B/r for 3 or 5 h, then nutrients were removed. NF cells were cells suspended in PB immediately after dissociation. The mixed cell suspension containing RF and NF cells (the ratio RF:NF = 15:85) at 1 to 2 × 10^6^ cells/mL was plated on a glass-bottomed dish (MatTek, Ashland, MA, USA) covered with a thin sheet of 1% PB agar. Confocal fluorescence images were taken using an inverted confocal microscope (Nikon A1+) and Z-slices were obtained using a Ti Z-drive.

### Real-time PCR analysis

Dissociated slug cells were suspended in each solution and shaken at 120 rpm before harvested at the selected time points. Quantitative PCR amplification (qPCR) was performed using a qPCR thermocycler (ABI7500, Applied Biosystems) with primer pairs and fluorescent beacons (Table S2). The levels of relative gene expression were calculated from the amplification value at the threshold cycle and the relative standard curve for each gene. And then, normalization was performed using *rnlA* amplification as an endogenous control.

### Statistical analysis

*P*-values from multiple comparisons were adjusted using Holm’s method. A generalized linear model (GLM) and analysis of deviance for the fit were performed for the analyses listed in Table S1.

### Supplementary Information

Supplementary Information 1.

Supplementary Information 2.

Supplementary Movie 1.

Supplementary Movie 2.

Supplementary Movie 3.

Supplementary Movie 4.

Supplementary Movie 5.

## Data Availability

The experimental data is included in Supplementary Material as Dataset S1.

## References

[1] Kessin RH (2001). Dictyostelium.

[2] Strassmann JE, Zhu Y, Queller DC (2000). Altruism and social cheating in the social amoeba *Dictyostelium discoideum*. Nature.

[3] Buttery NJ (2012). Structured growth and genetic drift raise relatedness in the social amoeba *Dictyostelium discoideum*. Biol. Lett..

[4] Kuzdzal-Fick JJ, Fox SA, Strassmann JE, Queller DC (2011). High relatedness is necessary and sufficient to maintain multicellularity in *Dictyostelium*. Science.

[5] Ho HI, Hirose S, Kuspa A, Shaulsky G (2013). Kin recognition protects cooperators against cheaters. Curr. Biol..

[6] Khare A (2009). Cheater-resistance is not futile. Nature.

[7] Katoh M, Chen G, Roberge E, Shaulsky G, Kuspa A (2007). Developmental commitment in *Dictyostelium discoideum*. Eukaryot. Cell.

[8] Raper KB (1940). Pseudoplasmodium formation and organization in *Dictyostelium discoideum*. J. Elisha Mitchell Sci. Soc..

[9] Katoh M (2004). An orderly retreat: Dedifferentiation is a regulated process. Proc. Natl. Acad. Sci. U.S.A..

[10] Gilbert OM, Queller DC, Strassmann JE (2009). Discovery of a large clonal patch of a social amoeba: Implications for social evolution. Mol. Ecol..

[11] Kuzdzal-Fick JJ, Foster KR, Queller DC, Strassmann JE (2007). Exploiting new terrain: An advantage to sociality in the slime mold *Dictyostelium discoideum*. Behav. Ecol..

[12] Chen G, Zhuchenko O, Kuspa A (2007). Immune-like phagocyte activity in the social amoeba. Science.

[13] Newell PC, Longlands M, Sussman M (1971). Control of enzyme synthesis by cellular interaction during development of the cellular slime mold *Dictyostelium discoideum*. J. Mol. Biol..

[14] Siu CH, Des Roches B, Lam TY (1983). Involvement of a cell-surface glycoprotein in the cell-sorting process of *Dictyostelium discoideum*. Proc. Natl. Acad. Sci. U.S.A..

[15] Siu CH, Harris TJC, Wang J, Wong E (2004). Regulation of cell–cell adhesion during *Dictyostelium* development. Semin. Cell Dev. Biol..

[16] Crowley TE, Nellen W, Gomer RH, Firtel RA (1985). Phenocopy of discoidin I-minus mutants by antisense transformation in *Dictyostelium*. Cell.

[17] Fujimori T, Nakajima A, Shimada N, Sawai S (2019). Tissue self-organization based on collective cell migration by contact activation of locomotion and chemotaxis. Proc. Natl. Acad. Sci. U.S.A..

[18] Steinberg MS, Takeichi M (1994). Experimental specification of cell sorting, tissue spreading, and specific spatial patterning by quantitative differences in cadherin expression. Proc. Natl. Acad. Sci. U.S.A..

[19] Parkinson K (2009). Regulation of Rap1 activity is required for differential adhesion, cell-type patterning and morphogenesis in *Dictyostelium*. J. Cell Sci..

[20] Finney RE, Mitchell LH, Soll DR, Murray BA, Loomis WF (1983). Loss and resynthesis of a developmentally regulated membrane protein (gp80) during dedifferentiation and redifferentiation in *Dictyostelium*. Dev. Biol..

[21] Lam TY, Pickering G, Geltosky J, Siu CH (1981). Differential cell cohesiveness expressed by prespore and prestalk cells of *Dictyostelium discoideum*. Differentiation.

[22] Whittingham WF, Raper KB (1960). Non-viability of stalk cells in *Dictyostelium*. Proc. Natl. Acad. Sci. U.S.A..

[23] Jermyn K, Traynor D, Williams J (1996). The initiation of basal disc formation in *Dictyostelium discoideum* is an early event in culmination. Development.

[24] Watts DJ, Treffry TE (1976). Culmination in the slime mould *Dictyostelium discoideum* studied with a scanning electron microscope. J. Embryol. Exp. Morphol..

[25] Frank SA (1995). Mutual policing and repression of competition in the evolution of cooperative groups. Nature.

[26] Queller DC, Ponte E, Bozzaro S, Strassmann JE (2003). Single-gene greenbeard effects in the social amoeba *Dictyostelium discoideum*. Science.

[27] Ponte E, Bracco E, Faix J, Bozzaro S (1998). Detection of subtle phenotypes: The case of the cell adhesion molecule csA in *Dictyostelium*. Proc. Natl. Acad. Sci. U.S.A..

[28] Bukahrova T (2005). Paxillin is required for cell-substrate adhesion, cell sorting and slug migration during *Dictyostelium development*. J. Cell Sci..

[29] Sternfeld J (1998). The anterior-like cells in *Dictyostelium* are required for the elevation of the spores during culmination. Dev. Genes Evol..

[30] Bichler G, Weijer CJ (1994). A *Dictyostelium* anterior-like cell mutant reveals sequential steps in the prespore prestalk differentiation pathway. Development.

[31] Thompson CRL, Kay RR (2000). Cell-fate choice in dictyostelium: Intrinsic biases modulate sensitivity to DIF signaling. Dev. Biol..

[32] Kuzdzal-Fick JJ, Queller DC, Strassmann JE (2010). An invitation to die: Initiators of sociality in a social amoeba become selfish spores. Biol. Lett..

[33] Deichsel H (1999). Green fluorescent proteins with short half-lives as reporters in *Dictyostelium discoideum*. Dev. Genes Evol..

