# Supplementary material for: Prestalk-like positioning of de-differentiated cells in the social amoeba Dictyostelium discoideum
Source: Sci Rep. 2024 Apr 1;14:7677. doi: 10.1038/s41598-024-58277-3 (PMC10985001; doi:10.1038/s41598-024-58277-3)
Supplement: Supplementary file 1 — Supplementary Information 1. [file 41598_2024_58277_MOESM1_ESM.pdf]

**Supplementary material**
**Supplementary Text and Figures for**

# Prestalk-like positioning of de-differentiated cells in the social amoeba *Dictyostelium discoideum*

Yuka Shirokawa*, Masakazu Shimada, Nao Shimada, Satoshi Sawai

*To whom correspondence should be addressed: Yuka Shirokawa
Email: shirokawa.yuka@gmail.com

**This PDF file includes:**

Supplementary text
Figures S1 to S5
Tables S1 and S2
Legends for Movies S1 to S5
Legends for Datasets S1
SI References

**Other electronic supplementary materials for this manuscript include the following:**

Movies S1 to S5
Datasets S1

# Supplementary Text

## (a) Strain construction

Cells were grown at 22°C in a shaking culture with PS growth medium[1]. To obtain cells that co-expressed prespore and prestalk fate markers, the following two constructs were introduced into AX4 cells and clones were isolated: (i) An RFPmars expression construct under the control of the prestalk-specific *ecmAO* promoter with G418 selection (ecmAO-RFP, Dicty Stock Center, plasmid ID 639), and (ii) A GFP expression construct under the control of the prespore-specific *pspA* promoter with hygromycin selection (pspA-GFP). For selection and plasmid maintenance, the growth medium containing 30 μg/mL G418 and 60 μg/mL hygromycin was used.

The pspA-GFP expression plasmid was constructed by replacing the *act15* promoter in pHygGFP[2] with the *pspA* promoter as follows: First, a *Cla*I restriction site in pHygGFP was replaced with a *Bgl*II site using the oligonucleotide linker 5'-CGACAGATCTGT-3'. Next, the *act15* promoter in the pHygGFP was replaced with the *pspA* promoter at *Xba*I and *Bgl*II sites. The *pspA* promoter

fragment was obtained from the pspA-Gal construct (Dicty Stock Center plasmid ID 49)[3] by excision at *Xba*I and *Bgl*II sites.

Strains constitutively expressing GFP or RFP were constructed by transforming AX4 with GFP or RFP expression constructs under the control of the *act15* promoter, with 10 µg/mL G418 selection. For GFP expression, pA15GFP (S65T)[4] was used. For RFP expression, pA15-mRFPmars[5] was used.

**(b) Refeeding experiments**

Vegetative cells were washed with phosphate buffer (PB) (pH 6.5; 20 mM $KH_2PO_4$, 20 mM $Na_2HPO_4$) and plated on a 1% PB agar (BactoAgar, Difco) plate at 0.3 to 0.4 $\times 10^6$ cells/cm$^2$ and incubated at 22°C for 18 h until the slug stage. The cells were mechanically dissociated in PB containing 20 mM EDTA (pH 6.4) through repeated pipetting and passage through a 23 G needle and a 40µm cell strainer, followed by washing with PB.

To prepare a bacterial suspension, *E. coli* B/r was grown in LB medium (10 g/L tryptone, 5 g/L yeast extract, and 10 g/L NaCl) until it reached an optical density (OD) of 3 at 600 nm. The culture was concentrated in PB at OD50 equivalent by centrifugation. The bacterial suspension was immediately used.

For refeeding, dissociated cells were co-suspended with *E. coli* B/r at OD50 in PB at a final density of 2 to 3 $\times 10^6$ cells/ml. The mixture was shaken for 3 or 5 h in a 50 ml centrifuge tube with a typical volume of 1.25 mL. The cells were then washed by pelleting and resuspending in PB. This washing procedure was repeated twice to remove bacteria. In the case of refeeding with growth media, the PS growth medium was used. NF cells were cells suspended in PB without nutrients immediately after dissociation.

For the mixing experiments (Figs. 1 and 2), the NF and RF cells were mixed in an 85:15 ratio. The mixed cell suspension adjusted to 1 to 2 $\times 10^6$ cells/mL was plated on a glass-bottomed dish (MatTek, Ashland, MA) covered with a thin sheet of 1% PB agar. The plates were left undisturbed for 15 min to allow the cells to attach to the agar before gently removing the buffer.

Confocal images of GFP, RFP, and calcofluor white fluorescence were captured using an inverted confocal microscope (A1+, Nikon), and Z-slices were obtained using Ti Z-drive. The cells were observed at three time windows after plating: 1.5–2 h for the aggregates with a 20$\times$ air objective lens, 4–5 h during early culmination for the upper part of a fruiting body with a 40$\times$ air objective lens, 8–10 h for the basal disk of terminally differentiated fruiting bodies with a 60$\times$ oil immersion objective lens, and for cells scattered around the base of fruiting bodies with a 20$\times$ air objective lens.

Images were analyzed using custom programs in ImageJ[6] and the R language[7]. To quantify cell localization, images were binarized according to the fluorescence of the cell fate markers. Lists of XY coordinates of the marker-positive pixels were obtained from the binarized images. For the analysis of cell aggregates, Z-sections were taken at 3 µm intervals between 30 to 60 µm from the bottom of the aggregate. The XY coordinates of each Z-slice were merged into a single list. The origin of the coordinates was set to the aggregate center, and the aggregate radius was normalized to 1. The average distance from the center to the marker-positive pixels was computed for the prestalk and prespore markers.

For the analysis of early culminants, the upper regions of fruiting bodies were attached to a glass slide, and Z-slices were acquired at 5 µm intervals from the bottom to the top of the sample. The lists of XY coordinates of marker-positive pixels from the Z-slices were merged. Each fruiting body was vertically aligned from the tip to the lower cup, and a normalized coordinate in the vertical direction was assigned from 0 to 1. The frequency of marker-positive pixels at each position was quantified for the prestalk and prespore markers. The vertical position was divided into 50 equally-spaced bins to obtain histograms.

For the analysis of the basal disk, stalks were transferred to a glass slide and stained with 0.01% calcofluor white (BD, NJ, USA). Basal disk regions were determined using calcofluor white fluorescence which stains cellulose from vacuolated stalk cells[8]. Z-section images were acquired at a 1.5µm interval from the bottom to the top of the sample. The number of marker-positive pixels in the region was acquired from binarized images, and the values from each slice were summed. The ratio of the pixel numbers between the prestalk and prespore images was calculated.

For cells scattered around the base of fruiting bodies, the fruiting body standing on an agar-coated glass-bottom dish was observed from the bottom of the dish. Z-stack images were captured from the bottom of the sample to a height of 30 µm with 5 µm intervals. The number of marker-positive pixels was acquired within a 100 µm radius from the center of the basal disk, and the values from each Z-slice were summed. Ratios between the values obtained from the prestalk and prespore images were calculated.

**(c) Quantitative real-time polymerase chain reaction analysis**

qRT-PCR analysis was performed as follows: For 'No-dissociation' sample (Fig. 1D), AX4 vegetative cells were starved on a 1% PB agar plate and harvested at the selected time points. For the other conditions ('No-nutrient buffer', 'Bacteria', 'Growth medium' in Fig. 1D), dissociated slug cells were either suspended in PB or in PB mixed with *E. coli* at OD50 or in the PS growth medium. The suspension was shaken in a 50 ml centrifuge tube. Cells were harvested at the selected time points. Total RNA was

extracted using a Maxwell 16 LEV simplyRNA cells and Tissue kit (Promega, WI, USA). cDNA was synthesized using random hexamers and SuperScript III (First-Strand Synthesis, Invitrogen). Quantitative PCR (qPCR) amplification was performed with a qPCR thermocycler (ABI7500, Applied Biosystems) using a pre-mixed reaction solution (TaqMan Universal PCR Master Mix, Applied Biosystems) and with primer pairs and fluorescent beacons (TaqMan probe MGB, Applied Biosystems) (Table S2). The amplification value at the threshold cycle (CT) of each sample was measured in three independent wells, and the average value was used. The levels of relative gene expression were calculated from the CT and the relative standard curves for each gene, followed by normalization using *rnlA* amplification as an endogenous control.

For hierarchical clustering of time series of the gene expression level, permutation distribution clustering was applied with R function 'pdc'. Time series from the three conditions ('No-nutrient buffer', 'Bacteria', and 'Growth medium') were embedded and the squared Hellinger distance was calculated to measure dissimilarity between gene targets. Subsequently, clustering was performed based on the dissimilarity. The results are presented in Fig. 1D.

To investigate temporal differences in gene expression levels, 'hypothesis testing with bootstrap'[9] was applied to the qRT-PCR data. Pair-wise comparisons were performed between the sampling time points 0h and 5h. The null hypothesis was that both the samples originated from the same probability distribution. We generated pairs of 100,000 bootstrap samples from a mixture of the observed data. The percentile rank of the observed *t*-values within the distribution of *t*-values was determined through the bootstrap sampling. And then, the percentile rank was used as the *P*-value for a two-sided test. The results are presented in Table S1.

**(d) Cell cohesiveness assay**

Cell cohesiveness was assayed as previously described[10] with minor modifications to the cell density and container geometry. Dissociated slugs with the cell fate markers were suspended at $6 \times 10^6$ cells/mL. The suspensions were placed in 50 mL tubes and shaken at 120 rpm for 1.5, 3, and 5 h. In the NF condition, cells were suspended in plain PB. In RF conditions, cells were suspended in PB with *E. coli* at OD50 or the PS growth medium. To quantify the number of cells not associated with aggregates, images of the samples loaded into a hemocytometer were acquired and binarized based on the fluorescence of the cell fate markers. The number of single cells was then calculated using the particle analyzer function in ImageJ. The results are presented in Fig. 1E.

**(e) Flow cytometry**

Dissociated cells with the cell fate markers were suspended in PB mixed with *E. coli* B/r at OD50 and shaken for 3, 6, and 12 h. The cells were washed with PB, and the intensity of RFP and GFP expression was measured using a flow cytometer (SH800, Sony, Japan). Cells that were negative for ecmAO-RFP fluorescence and positive for pspA-GFP fluorescence were identified as 'Prespore fate', whereas cells that were positive for ecmAO-RFP fluorescence and negative for pspA-GFP fluorescence were categorized as 'Prestalk fate'. Cells that were negative for both ecmAO-RFP and pspA-GFP fluorescence were identified as 'Non-fluorescent cells'. The results are presented in Fig. S5.

**(f) Terminal cell fate allocation within RF and NF cell mixture**

In the terminal fate (spores and solitary cells), we quantified the ratio of RF cells to NF cells. For NF and RF cells, we utilized cells expressing GFP or RFP constitutively under the strong actin15 promoter. The NF and RF cells were mixed at 1:1 ratio. The cell mixtures were applied on a 1% PB agar plate. After incubating at 22°C for 10 h, when the development of the fruiting body had more or less completed, PB containing 20 mM EDTA was poured onto the agar plate, and spores and solitary amoeboid cells were harvested by pipetting. The cell suspension was treated with 0.01% calcofluor white (BD, NJ, USA) to distinguish spores from amoeboid cells[8]. The identification of RF cells from NF cells is based on the expression of GFP and RFP. Images of the samples loaded into a hemocytometer were acquired, and binarized based on the fluorescence, and the number of cells was calculated using the particle analyzer function in ImageJ. For the illustration in Fig. S4A, the GFP-RFP ratio in the terminal state was adjusted using the ratio of GFP-RFP in cells immediately after mixing, and then normalized by the control mixture NF(GFP)/NF(RFP). Additionally, Fig. S4B presents the data without adjustment and normalization for interpretation.

**(h) Statistical analysis**

All statistical analyses were performed using the R software[7]. Details of a generalized linear model (GLM) and analysis of deviance for the fit are listed in Table S1. For multiple comparisons, the *P*-value was adjusted using Holm's method.

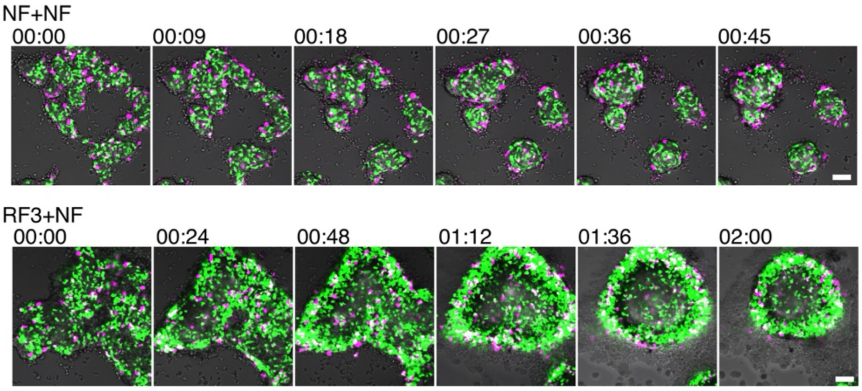


**Figure S1 The time course of the reaggregation of dissociated cells.**

Time-series of the development following the plating of cell mixtures (NF+NF or RF+NF) are presented, capturing images from the moment of plating (0:00) until the characteristic sorting pattern emerges. The quantification of cell positioning (Fig. 1 B-C) was based on aggregations 1.5–2 hours after plating, consistent across both the NF+NF and RF+NF conditions. The merged fluorescent and bright field images. In the NF+NF condition, prestalk cells (ecmAO-RFP, magenta) were sorted to the peripheral region of the aggregate, whereas prespore cells (pspA-GFP, green) were more uniformly distributed. In the RF3+NF condition, RF cells of both prestalk and prespore origins were well mixed and subsequently sorted out to the periphery of the aggregates. The numbers on the upper left side indicate hour:min. All scale bars = 50 µm.

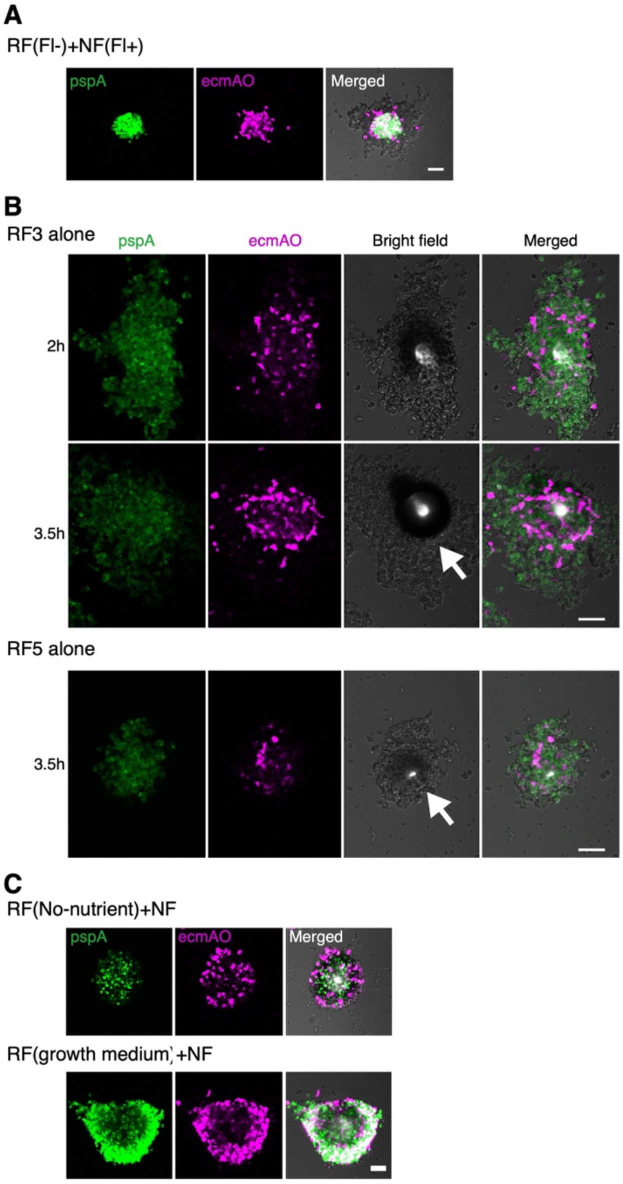


**Figure S2 Supporting data for refeeding experiments.**

**(A)** The swap control. NF cells with the cell-type reporter (fluorescent) and nonfluorescent RF3 cells were mixed at a ratio 15:85 (hereafter referred to as RF(Fl-)+NF(Fl+)). Representative images of mixed aggregates (pspA: GFP-channel. ecmAO: RFP-channel. Merged: overlay of bright-field (grayscale) and fluorescence images). A reciprocal pattern of fluorescent cells in the center and non-fluorescent cells in the periphery were shown. The result indicates that expression of the marker genes itself did not affect the sorting pattern.

**(B)** RF cells were plated alone on the non-nutrient agar and their aggregation was observed. '2h' and '3.5h' indicate the time after plating. 'RF3 alone' and 'RF5 alone' indicate RF3 or RF5 cells plated alone, respectively. Prestalk cells were found in the peripheral region of the aggregate, while prespore cells were more uniformly distributed. Furthermore, RF cells formed a mound and tip (arrow) at roughly

the same time as NF cells. These results indicate that RF cells were still fully capable of developing on their own. Therefore, the segregation between NF and RF cells in aggregates was not due to the innate inability of RF cells to develop, but rather a consequence of their relative behavior when in association with the NF cells.

**(C)** Experiments to show the requirement of nutrient replenishment for the segregation. RF(No-nutrient)+NF: Cells with fluorescent markers were shaken for 3 h in the plain phosphate buffer, and mixed with nonfluorescent NF cells. The result indicates that cells shaken in the non-nutrient buffer were not segregated from the freshly dissociated cells.

RF(growth medium)+NF: Cells with fluorescent were shaken for 3 h in the growth medium, and mixed with nonfluorescent NF cells. Refed cells with growth medium were sorted to the peripheral region, whereas NF cells were more uniformly distributed. The result suggests that the segregation pattern of refeeding with growth medium is the same as that of refeeding with bacteria.

Taken together, these experiments indicate that the unique sorting pattern was not merely due to mechanical interruption but also required nutrient replenishment. All scale bars = 50 µm.

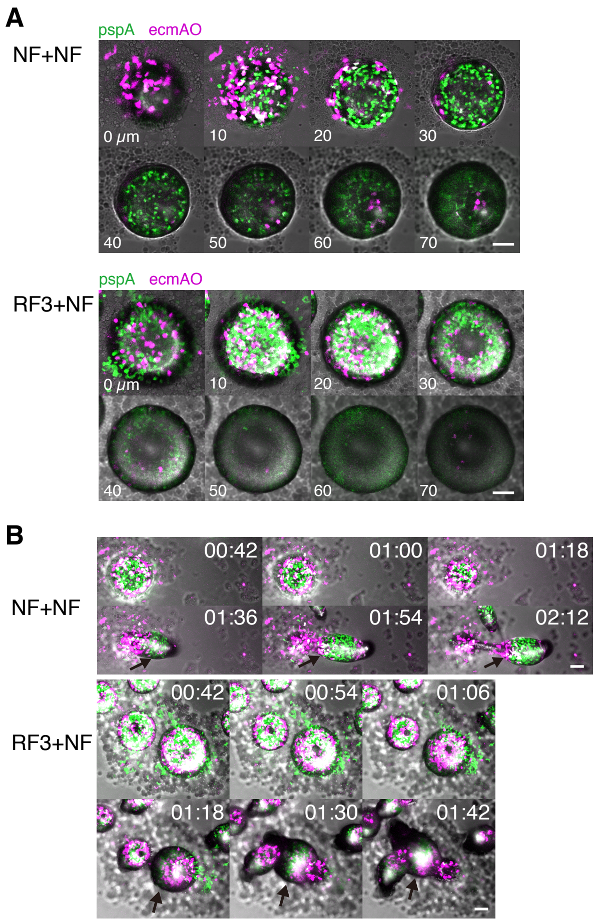


**Figure S3 Fruiting body formation of refed cells.**

**(A)** Z stack images of an aggregate in the tip formation stage (3h after plating). Upper panels: NF+NF. Lower panels: RF3+NF. The numbers in the images are the height from the bottom of an aggregate (µm). In the NF+NF condition, prestalk cells localized to the bottom of the aggregate, while prespore cells were found in the upper regions. In the RF3+NF condition, RF cells of both prestalk and prespore origins localized to the bottom.

**(B)** The time series of fruiting body formation. The maximum intensity projections of Z-stack images are shown. In the NF+NF condition, NF prespore cells occupied the middle region of the fruiting body (arrows). In the RF3+NF condition, RF cells localized to the lower cup region of the fruiting body. These results indicate that RF prespore cells lost their ability to occupy the upper region of the fruiting body. The numbers on the upper right side represent hour:min, with 0:00 indicating the starting point of imaging during the aggregation phase. The time points were chosen to highlight significant cell dynamics, such as culmination and cell positioning. For a comprehensive view of the dynamics, see Movie S3 and S4. The quantification of cell positioning (Fig. 2B) was based on fruiting bodies observed 4-5h after plating, consistent across both the NF+NF and RF+NF conditions. All scale bars = 50 µm.

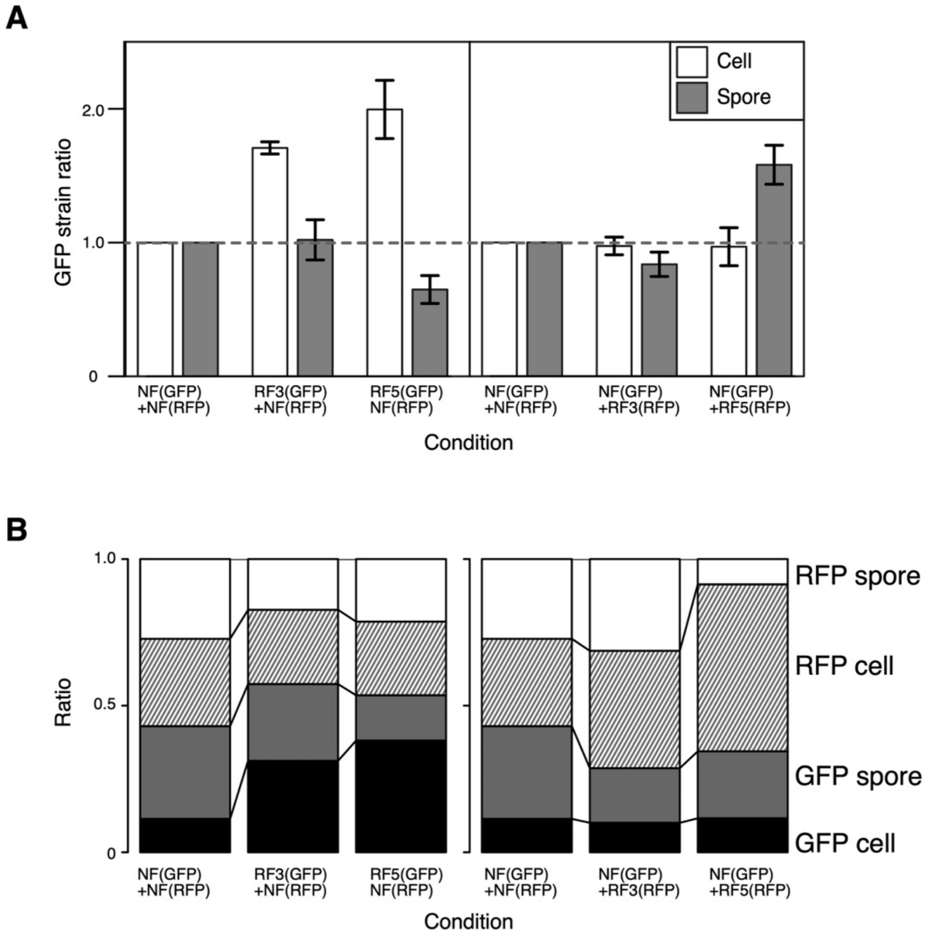


**Figure S4 Terminal cell fate allocation in the mixed cell population.**

**(A)** GFP strain ratio in the cell mixtures with a normalization. NF and RF cells constitutively expressed GFP or RFP. The mixture of NF GFP cells and NF RFP cells (NF(GFP)/NF(RFP)) is the control mixture. Left panel: The mixtures of RF GFP cells with NF RFP cells, denoted as (RF3(GFP)/NF(RFP), RF5(GFP)/NF(RFP)). Right panel: The mixtures of RF RFP cells with NF GFP cells, denoted as (NF(GFP)/RF3(RFP), NF(GFP)/RF5(RFP)). For the illustration, the GFP ratio was adjusted using the ratio of GFP-RFP in cells immediately after mixing, and then normalized by the control mixture NF(GFP)/NF(RFP). Error bars: Standard error.

**(B)** Cell ratio in the cell mixtures without the adjustment and normalization. The ratio represents the average of three biological replicates.

Despite our strict control over experimental conditions, the experiments tend to exhibit slightly larger variation, likely due to the dissociation, refeeding, and initiation of growth in RF solitary cells. Additionally, although strains constitutively expressing GFP or RFP were utilized, there was a tendency for slightly less basal production of spores and solitary cells in the GFP strain. Despite these challenges, at least, the results indicate that both RF(GFP) and RF(RFP) exhibited proportional changes compared

to NF cells. In the statistical analysis, the crude count data in the terminal state was used as response variable, and the ratio of cells with GFP toward RFP in cells of immediately after mixing was used as an offset term to adjust the response variable. In the RF+NF, RF cells were redirected into a higher proportion of solitary amoeboid cells than NF cells (GLMM and analysis of deviance, $P < 0.0001$, Table S1). On the other hand, NF cells differentiated into a higher proportion of spore than RF cells (GLMM and analysis of deviance, $P < 0.0001$, Table S1). Sample size N represents three biological replicates with at least 3600 cells (amoeboid cells and spores) per condition.

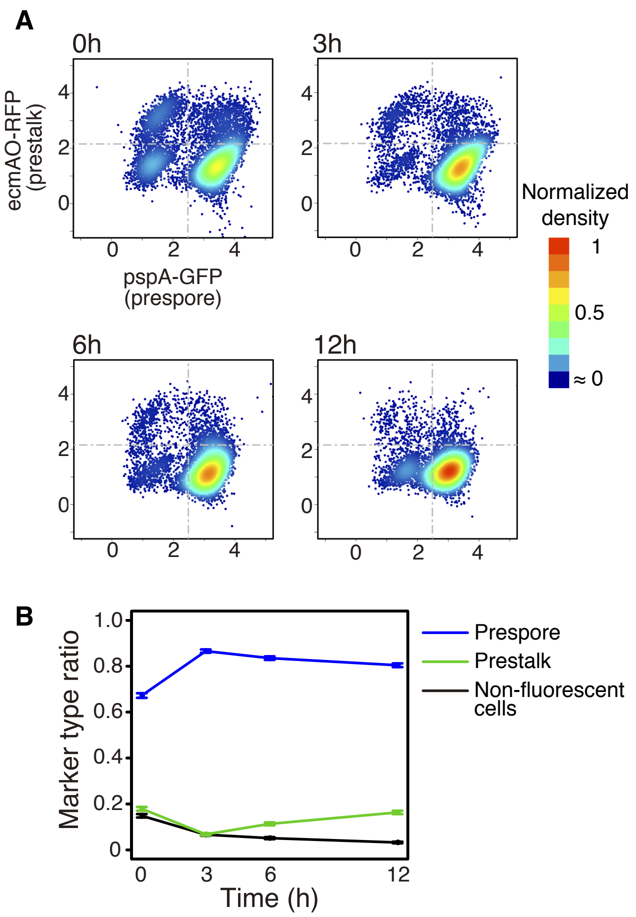


**Figure S5 Flow cytometry analysis of cells during refeeding.**

**(A)** Scatter plot showing fluorescent intensities of cell fate markers ecmAO-RFP (prestalk) and pspA-GFP (prespore). Cells from dissociated slugs were incubated in the bacterial suspension.

**(B)** Temporal changes in the ratio of cell types. Error bars: 95% confidence interval (CI).

**Table S1** Summary of statistical analysis. GLM is generalized linear model. $\beta_0$ , $\beta_1$ and $\beta_2$ indicate regression coefficients. Rv: Response variable, Ev: Explanatory variable. Link: Link functions that decide relationship between Rv and Ev. * indicates $P$ <0.05

| **Analysis** | **Cell localization within aggregations for Fig. 1C** |
|---|---|
| **Model formula**<br>**R code format**<br>**Explanation** | $g(d) = \beta_0 + \beta_1\, condition + \beta_2\, cellfate.$<br>$glm(d{\sim}condition + cellfate, family = gaussian(link = "identity")).$<br>GLM with gaussian error. Rv: $d$, Ev: $condition$ and $cellfate$. Link: $g(d) = d$.<br>$d$: The mean distance from center of a mound to each pixel. $condition$: Experimental condition, $cellfate$: Prespore or prestalk. |
| **Results** | **Overall model:** GLM and analysis of deviance, Cell fate: $F_{1,186}$ = 228.661, $P$ < 0.0001*, Conditions: $F_{2,184}$ = 56.447, $P$ < 0.0001*<br>**Multiple comparison:** $t$-test<br>1: Prespore (NF+NF) vs Prestalk (NF+NF): d.f. = 65.658, t = -14.222, adjusted $P$ < 0.0001*<br>2: Prespore (NF+NF) vs Prespore (RF3+NF): d.f. = 65.472, t = -8.1747, adjusted $P$ < 0.0001*<br>3: Prespore (NF+NF) vs Prestalk (RF3+NF): d.f. = 57.518, t = -16.525, adjusted $P$ < 0.0001*<br>4: Prespore (NF+NF) vs Prespore (RF5+NF): d.f. = 36.687, t = -7.606, adjusted $P$ < 0.0001*<br>5: Prespore (NF+NF) vs Prestalk (RF5+NF): d.f. = 39.133, t = -14.512, adjusted $P$ < 0.0001*<br>6: Prestalk (NF+NF) vs Prespore (RF3+NF): d.f. = 63.852, t = 4.4839, adjusted $P$ < 0.001*<br>7: Prestalk (NF+NF) vs Prestalk (RF3+NF): d.f. = 55.146, t = -6.1947, adjusted $P$ < 0.0001*<br>8: Prestalk (NF+NF) vs Prespore (RF5+NF): d.f. = 35.066, t = 1.348, adjusted $P$ = 0.373<br>9: Prestalk (NF+NF) vs Prestalk (RF5+NF): d.f. = 37.259, t = -4.973, adjusted $P$ < 0.0001*<br>10: Prespore (RF3+NF) vs Prestalk (RF3+NF): d.f. = 63.301, t = -9.1856, adjusted $P$ < 0.0001*<br>11: Prespore (RF3+NF) vs Prespore (RF5+NF): d.f. = 41.336, t = -1.7063, adjusted $P$ = 0.286<br>12: Prespore (RF3+NF) vs Prestalk (RF5+NF): d.f. = 44.369, t = -7.856, adjusted $P$ < 0.0001*<br>13: Prestalk (RF3+NF) vs Prespore (RF5+NF): d.f. = 50.541, t = 5.7015, adjusted $P$ < 0.0001*<br>14: Prestalk (RF3+NF) vs Prestalk (RF5+NF): d.f. = 53.52, t = 0.60077, adjusted $P$ < 0.551<br>15: Prespore (RF5+NF) vs Prestalk (RF5+NF): d.f. = 47.565, t = -4.8928, adjusted $P$ < 0.0001* |
| **Analysis** | **Quantitative real-time polymerase chain reaction analysis for Fig. 1D**<br>Analysis to detect nutrient-specific changes |
| **Model formula**<br>**R code format**<br>**Explanation** | $g(q) = \beta_0 + \beta_1\, condition.$<br>$glm(q{\sim}condition, family = gaussian(link = "identity")).$<br>GLM with gaussian error. Rv: $q$, Ev: $condition$, Link: $g(q) = q$.<br>$q$: The levels of relative gene expression.<br>$condition$: Experimental condition. |
| **Results** | To understand cell-state change incurred by refeeding, following comparisons are considered.<br>**Multiple comparison:** GLM and analysis of deviance<br>No-nutrient versus Bacteria:<br>emcA: $F_{1,16}$ = 0.1013, adjusted P = 1<br>pspA: $F_{1,16}$ = 0.2646, adjusted P = 1<br>acaA: $F_{1,16}$ = 7.2018, adjusted P = 0.01631*<br>carA: $F_{1,16}$ = 15.289, adjusted P = 0.001247*<br>pdsA: $F_{1,16}$ = 14.177, adjusted P = 0.003385*<br>dscA: $F_{1,16}$ = 13.907, adjusted P = 0.001981*<br>tgrC: $F_{1,16}$ = 1.0826, adjusted P = 0.627171<br>cadA: $F_{1,16}$ = 0.1726, adjusted P = 1<br>csaA: $F_{1,16}$ = 0.1726, adjusted P = 1<br>No-nutrient versus Growth medium:<br>emcA: $F_{1,16}$ = 0.4288, adjusted P = 1<br>pspA: $F_{1,16}$ = 0.0279, adjusted P = 1<br>acaA: $F_{1,16}$ = 12.666, adjusted P = 0.005232*<br>carA: $F_{1,16}$ = 24.939, adjusted P = 0.000265*<br>pdsA: $F_{1,16}$ = 11.535, adjusted P = 0.003689*<br>dscA: $F_{1,16}$ = 16.156, adjusted P = 0.001981*<br>tgrC: $F_{1,16}$ = 0.5538, adjusted P = 0.627171<br>cadA: $F_{1,16}$ = 0.0767, adjusted P = 1<br>csaA: $F_{1,16}$ = 0.0767, adjusted P = 1 |

The continuation of Table S1.

| **Analysis** | **Quantitative real-time polymerase chain reaction analysis for Fig. 1D**<br>Analysis to detect temporal differences in the levels of gene expression |
|---|---|
| **Explanation** | Pair-wise comparisons of levels of relative gene expression were performed between the sampling time points 0h and 5h with the bootstrap method. For the detail, see SI text, ‘Quantitative real-time polymerase chain reaction analysis’. |
| **Results** | Bootstrap method<br>No-nutrient buffer 0h vs 5h<br>emcA: t = 2.243471, *P* = 0.02881<br>pspA: t = 2.570983, *P* = 0.02438**<br>acaA: t = -0.8896212, *P* = 0.21216<br>carA: t = -13.71146, *P* = 0.01272**<br>pdsA: t = -13.44524, *P* = 0.01206**<br>dscA: t = -0.9597171, *P* = 0.1883<br>tgrC: t = 4.070634, *P* = 0.01408**<br>cadA: t = 3.525237, *P* = 0.01524**<br>csaA: t = -4.731203, *P* = 0.0121**<br>Bacteria 0h vs 5h<br>emcA: t = 6.490245, *P* = 0.01275**<br>pspA: t = 6.184117, *P* = 0.01264**<br>acaA: t = 1.342081, *P* = 0.12785<br>carA: t = 0.9833843, *P* = 0.18435<br>pdsA: t = 1.421683, *P* = 0.1052<br>dscA: t = 3.987957, *P* = 0.01547**<br>tgrC: t = 0.9550938, *P* = 0.19265<br>cadA: t = 1.30704, *P* = 0.13153<br>csaA: t = -2.370863, *P* = 0.03002<br>Growth medium 0h vs 5h<br>emcA: t = 5.054192, *P* = 0.01153**<br>pspA: t = 4.0807, *P* = 0.01505**<br>acaA: t = 6.217846, *P* = 0.01101**<br>carA: t = 5.886053, *P* = 0.0126**<br>pdsA: t = 1.348934, *P* = 0.11539<br>dscA: t = 2.10459, *P* = 0.03932<br>tgrC: t = 8.379845, *P* = 0.00888**<br>cadA: t = 2.570532, *P* = 0.02075**<br>csaA: t = 7.610438, *P* = 0.01291**<br>No-dissociation 0h vs 5h<br>emcA: t = -2.934227, *P* = 0.01913**<br>pspA: t = 1.194258, *P* = 0.14051<br>acaA: t = 0.8418263, *P* = 0.24907<br>carA: t = 1.136168, *P* = 0.15566<br>pdsA: t = 0.007316311, *P* = 0.4876<br>dscA: t = 4.799018, *P* = 0.01373**<br>tgrC: t = 0.08839139, *P* = 0.5383<br>cadA: t =1.56493, *P* = 0.07794<br>csaA: t = 0.1677567, *P* = 0.43407<br><br>** indicates *P* <0.025.<br>The *p*-value was the percentile rank of the observed *t*-values within bootstrap sampling data. For a two-sided test, *P* <0.025 was used for the significance. |

The continuation of Table S1.

| Analysis | Number of single cells in aggregation assay for Fig. 1E (left) |
|---|---|
| **Model formula**<br>**R code format**<br>**Explanation** | $g(single) = \beta_0 + \beta_1\, condition.$<br>$glm(single{\sim}condition, family = poisson(link = "log")).$<br>GLM with poisson error. Rv: $single$, Ev: $condition$, Link: $g(single) = log(single)$. $single$: The number of single-state cells. $condition$: Experimental condition. |
| **Results** | To understand cell cohesion change incurred by refeeding, following comparisons are considered.<br>**Multiple comparison:** GLM and analysis of deviance<br>Buffer 5 h versus Bacteria 5 h<br>deviance = 2612.3, residual deviance = 424.93, d. f. = (1, 4), adjusted $P < 0.0001$*<br>Buffer 5 h versus Growth medium 5 h<br>deviance = 5920.9, residual deviance = 748.4, d. f. = (1, 4), adjusted $P < 0.0001$* |
| **Analysis** | **Proportion of single prespore cells in aggregation assay for Fig. 1E (right)** |
| **Model formula**<br>**R code format**<br>**Explanation** | $g(P_{prespore}) = \beta_0 + \beta_1\, condition.$<br>$glm(cbind(prespore, prestalk){\sim}condition, family = binomial(link = \text{"cloglog"})).$<br>GLM with binomial error. Rv: $P_{prespore}$, Ev: $condition$, Link: $g(P_{prespore}) = log(1 - log(1 - P_{prespore}))$. $P_{prespore}$: Probability that single state cells were expressed prespore marker. $prespore$: Prespore cell number. $prestalk$: Prestalk cell number. $condition$: Experimental condition. |
| **Results** | To understand the effect of refeeding on prespore ratio in the unattached cells, the following comparisons are considered.<br>**Multiple comparison:** GLM and analysis of deviance<br>Buffer+EDTA versus Buffer 5 h<br>deviance = 133.4, residual deviance = 9.438, d.f. = (1,4), adjusted $P < 0.0001$*<br>Buffer+EDTA versus Bacteria 5 h<br>deviance = 0.31927, residual deviance = 13.964, d.f. = (1, 4), adjusted $P = 0.572$<br>Buffer+EDTA versus Growth medium 5 h<br>deviance = 7.228, residual deviance = 17.483, d.f. = (1,4), adjusted $P = 0.01435508$*<br>Buffer 5 h versus Bacteria 5 h<br>deviance = 131.66, residual deviance = 9.073, d.f. = (1,4), adjusted $P < 0.0001$*<br>Buffer 5 h versus Growth medium 5 h<br>deviance = 159.01, residual deviance = 12.592, d.f. = (1,4), adjusted $P < 0.0001$* |
| **Analysis** | **Cell localization in an upper part of a fruiting body for Fig. 2B.** |
| **Model formula**<br>**R code format**<br>**Explanation** | $g(fspore) = \beta_0 + \beta_1\, fstalk.$<br>$glm(fspore{\sim}fstalk, family = Gamma(link = "log")).$<br>GLM with gamma error. Rv: $fspore$, Ev: $fstalk$. Link: $g(fspore) = log(fspore)$.<br>$fspore$: Prespore frequency at each vertical position in a fruiting body. $fstalk$: Prestalk frequency at the corresponding vertical position. To use log link function, we added 0.1 to the score of $fspore$ and $fstalk$ before analysis, because these included a score 0. |
| **Results** | No-dissociation: slope =-4.69333 ± 0.28704, $P < 0.0001$*<br>NF+NF: slope = -2.03408 ± 0.26450, $P < 0.0001$*<br>RF3+NF: slope = 4.52924 ± 0.24770, $P < 0.0001$*<br>RF5+NF: slope =3.69829 ± 0.30505, $P < 0.0001$* |
| **Analysis** | **Prespore marker expression ratio in a basal disk for Fig. 2E.** |
| **Model formula**<br>**R code format**<br><br>**Explanation** | $g(Iprespore) = \beta_0 + \beta_1\, condition + log(Iprestalk).$<br>$glm(Iprespore{\sim}condition, offset = log(Iprestalk), family = gaussian(link = "identity")).$<br>GLM with gaussian error. Rv: $Iprespore$, Ev: $condition$, Link: $g(Iprespore) = Iprespore$. $Iprespore$: Intensity value of prespore cell marker expression, $Iprestalk$: Intensity value of prestalk marker expression, $condition$: Experimental condition. $log(Iprestalk)$ is an offset term to adjust $Iprespore$ toward $Iprestalk$. |
| **Results** | **Overall model:** GLM and analysis of deviance, Conditions: $F_{3,94} = 25.008$, $P < 0.0001$*<br>**Multiple comparison:** $t$-test<br>NF+NF versus No-dissociation: d.f. = 53.998, t = 1.9158, adjusted $P = 0.06069$<br>NF+NF versus RF3+NF: d.f. = 22.468, t = -9.1623, adjusted $P < 0.0001$*<br>NF+NF versus RF5+NF: d.f. = 18.888, t = -12.292, adjusted $P < 0.0001$* |

The continuation of Table S1.

| Analysis | **Prespore ratio among cells left behind in basal region for Fig. 2G.** |
|---|---|
| **Explanation** | Same as the model formula for "Prespore marker expression ratio in a basal disk for Fig. 2E". |
| **Results** | **Overall model:** GLM and analysis of deviance, Conditions: F3,86 = 41.432, P < 0.0001*<br>**Multiple comparison:** *t*-test<br>NF+NF versus No-dissociation: d.f. = 43.841, t = 3.7784, adjusted *P* < 0.001*<br>NF+NF versus RF3+NF: d.f. = 23.08, t = -7.2017, adjusted *P* < 0.0001*<br>NF+NF versus RF5+NF: d.f. = 23.099, t = -21.81, adjusted P < 0.0001* |
| **Analysis** | **Allocation between social and solitary fitness for Fig. S4.** |
| **Model formula**<br>**R code format**<br><br>**Explanation** | $g(P_{gfp}) = \beta_0 + \beta_1\, condition + log(P_{initial}) + r.$<br>$glmer(cbind(gfp, rfp) \sim condition + (1\|r), offset = log(P_{initial}), family = binomial(link = "cloglog")).$<br>GLMM with binomial error. Rv: $P_{gfp}$, Ev: $condition$, Link: $g(P_{gfp}) = log(1 - log(1 - P_{gfp}))$. $P_{gfp}$: Probability of cells (or spores) expressed constitutive GFP in terminal differentiation. $gfp$: GFP cell number, $rfp$: RFP cell number, $condition$: Experimental condition, $P_{initial}$: Ratio of cells with constitutive GFP toward RFP in cells of immediately after mixing. $log(P_{initial})$ is an offset term to adjust $P_{gfp}$ by $P_{initial}$. $r$ is the difference by replicate experiments as a random effect. |
| **Results** | GLMM and analysis of deviance<br>For Fig. S4, *left,*<br>Conditions: (NF(GFP)/NF(RFP)), (RF3(GFP)/NF(RFP)), (RF5(GFP)/NF(RFP))<br>Effect of condition on probability of GFP cells, deviance = 189.41, d.f. = (4, 2), *P* < 0.0001*<br>Effect of condition on probability of GFP spores, deviance = 368.41, d.f. = (4, 2), *P* < 0.0001*<br><br>For Fig. S4, *Right,*<br>Conditions: (NF(GFP)/NF(RFP)), (NF(GFP)/RF3(RFP)), (NF(GFP)/RF5(RFP))<br>Effect of condition on probability of GFP cells, deviance = 133.32, d.f. = (4,2), *P* <0.0001*<br>Effect of condition on probability of GFP spores, deviance = 336.84, d.f. = (4,2), *P* <0.0001* |

**Table S2** Primer pairs and fluorescent beacons for qRT-PCR. * indicates primers and beacons that are newly constructed in this study. The rest are previously described[11].

| Gene name | | 5' →3' | Location | | Reporter Dye |
|---|---|---|---|---|---|
| *rnlA* | Forward | CGGATAAAAGGTACGCTAGGGATA | 2327 | 2350 | - |
| | Reverse | GTGCCGAACCACATAACAGATATG | 2375 | 2398 | - |
| | Probe | CAGGCTAGTCACATATT | 2352 | 2368 | VIC |
| *acaA* | Forward | TTGGTATTAGTCATGGTCCTTTGG | 3833 | 3856 | - |
| | Reverse | GAGGCGGTATTGGCAGTATCA | 3903 | 3923 | - |
| | Probe | CTGGTTGTATCGGTATCAG | 3860 | 3878 | FAM |
| *carA* | Forward | AAATATGTTTCCACCAGCACTCAA | 693 | 716 | - |
| | Reverse | GATAAATGTGACAGATGCCCAAAA | 751 | 774 | - |
| | Probe | ATTCTCCACACCTATTTG | 718 | 735 | FAM |
| *pdsA* | Forward | AGCAAGTGGCATTGAATATCCA | 789 | 807 | - |
| | Reverse | ACCAAAGACATAGTGGTGGCATT | 829 | 851 | - |
| | Probe | TCACAGAGTTGGTCCC | 809 | 824 | FAM |
| *tgrC1* | Forward | CCTCCAACACCAATAGATGCAA | 64 | 85 | - |
| | Reverse | GTTCTGGGTTCTTTTTCGTTTTTATACA | 152 | 179 | - |
| | Probe | TAATAGTAATCTCCCATATTCTACC | 117 | 141 | FAM |
| *ecmA* | Forward | GTTAATGCGGAAACTGAAACCA | 58 | 79 | - |
| | Reverse | CAAAAAGTAAACCTGCAGAACACA | 146 | 169 | - |
| | Probe | ACAAACCAATACAGCATGTG | 81 | 100 | FAM |
| *pspA* | Forward | GCGCTGATCAAACTTCTTCACAT | 263 | 285 | - |
| | Reverse | GGGTGTGGCAGTGATTTTACAA | 312 | 333 | - |
| | Probe | CACTCGGTTCTGATTGG | 287 | 303 | FAM |
| *dscA* | Forward | GGTCGTGGTGATGCTGATCA | 232 | 251 | - |
| | Reverse | CGATATTCAAACCAGGAAACATTATC | 286 | 311 | - |
| | Probe | TACATCATACAAAATCCG | 258 | 275 | FAM |
| *cadA*[*] | Forward | AATTGGCTCAAGGCAGTACAAAC | 209 | 231 | - |
| | Reverse | AAAAGCTCCTGGTAAGACTTGGAA | 265 | 288 | - |
| | Probe | TAACCTCAATAAATGGTCTTTC | 239 | 260 | FAM |
| *csaA*[*] | Forward | AACAATTTATTTCCTCGTGCCAAA | 462 | 485 | - |
| | Reverse | TTGAAAAGCCAAATGGTTGAATG | 519 | 541 | - |
| | Probe | CAATCGCTGGTGGTCTA | 488 | 504 | FAM |

**Movie S1 (separate file)** The process of aggregation formation in NF+NF condition. Images were obtained every 12 s. For the explanation, see Fig. S1. Scale bars = 50 µm.

**Movie S2 (separate file)** The process of aggregation formation in RF3+NF condition. Images were obtained every 12 s. For the explanation, see Fig. S1. Scale bars = 50 µm.

**Movie S3 (separate file)** The process of fruiting body formation in NF+NF condition. Images were obtained every 3 min. For the explanation, see Fig. S3B. Scale bars = 50 µm.

**Movie S4 (separate file)** The process of fruiting body formation in RF3+NF condition. Images were obtained every 3 min. For the explanation, see Fig. S3B. Scale bars = 50 µm.

**Movie S5 (separate file)** Solitary cells left behind in the base of the fruiting body in RF5+NF condition. Some moving cells were observed in the base 7 h after plating. Images were obtained every 10 s. The numbers on the upper right side indicate min:sec. Scale bars = 50 µm.

**Dataset S1 (separate file)** Data for the experiments.

## SI References


1. Sawai, S., Guan, X. J., Kuspa, A. & Cox, E. C. High-throughput analysis of spatio-temporal dynamics in *Dictyostelium*. *Genome Biol* **8**, R144 (2007).

2. Taniguchi, D. *et al.* Phase geometries of two-dimensional excitable waves govern self-organized morphodynamics of amoeboid cells. *Proc Natl Acad Sci USA* **110**, 5016–5021 (2013).

3. Dingermann, T. *et al.* Optimization and in situ detection of *Escherichia coli* β-galactosidase gene expression in *Dictyostelium discoideum*. *Gene* **85**, 353–362 (1989).

4. Fey, P., Compton, K. & Cox, E. C. Green fluorescent protein production in the cellular slime molds *Polysphondylium pallidum* and *Dictyostelium discoideum*. *Gene* **165**, 127–130 (1995).

5. Masaki, N., Fujimoto, K., Honda-Kitahara, M., Hada, E. & Sawai, S. Robustness of self-organizing chemoattractant field arising from precise pulse induction of its breakdown enzyme: A single-cell level analysis of PDE expression in *Dictyostelium*. *Biophys J* **104**, 1191–1202 (2013).

6. Abràmoff, M. D., Magalhães, P. J. & Ram, S. J. Image Processing with ImageJ. *Biophotonics International* **11**, 36–42 (2004).

7. *R: A Language and Environment for Statistical Computing. R Foundation for Statistical Computing, Vienna, Austria. URL Https://Www.R-Project.Org/.* (2015).

8. Harrington, B. J. & Raper, K. B. Use of a fluorescent brightener to demonstrate cellulose in the cellular slime molds. *Appl Microbiol* **16**, 106–113 (1968).

9. Efron, B. & Tibshirani, R. J. *An Introduction to the Bootstrap*. (Chapman & Hall, New York, 1993).

10. Parkinson, K. *et al.* Regulation of Rap1 activity is required for differential adhesion, cell-type patterning and morphogenesis in *Dictyostelium*. *J Cell Sci* **122**, 335–344 (2009).

11. McQuade, K. J., Nakajima, A., Ilacqua, A. N., Shimada, N. & Sawai, S. The green tea catechin epigallocatechin gallate (EGCG) blocks cell motility, chemotaxis and development in *Dictyostelium discoideum*. *PLoS ONE* **8**, e59275 (2013).
